# Supplementary material for: Identification of photoperiod-induced specific miRNAs in the adrenal glands of Sunite sheep (Ovis aries)
Source: Front Vet Sci. 2022 Jul 22;9:888207. doi: 10.3389/fvets.2022.888207 (PMC9354845; doi:10.3389/fvets.2022.888207)
Supplement: Supplementary file 1 [file Data_Sheet_1.ZIP › Supplementary file/miRDeep2 file.zip!/miRDeep2 file/Know_Structure/SP42A2/oar-mir-21.pdf]

miRBase precursor : oar-mir-21
Total read count : 291827
oar-miR-21 read count : 291808
remaining reads : 19

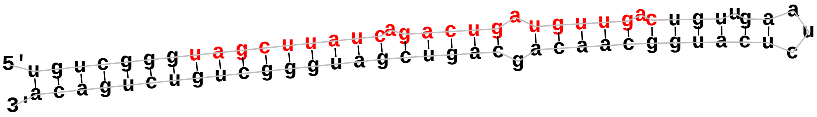

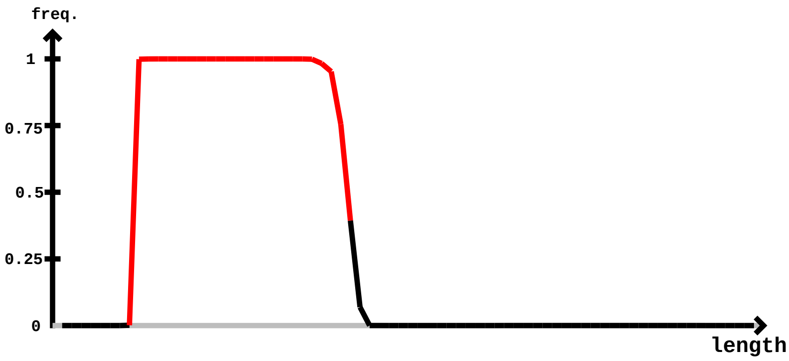

oar-miR-21

5'- ugucgggu**agcuuaucagacugauguugac**uguugaaucucauggcaacagcagucgaugggcugucugaca -3' exp

(((((((((((((((((.(((((.(((((.((((.((...))))))))))).))))))))))))))))))))))	reads	mm	sample
......guagcuuaucagacugaugu..	10	0	seq
......guagcuuaucagacugauguu...	28	0	seq
......guagcuuaucagacugauguug..	134	0	seq
......guagcuuaucagacugauguuga...	142	0	seq
......guagcuuaucagacugauguugac..	32	0	seq
......guagcuuaucagacugauguugacu...	14	0	seq
.......uagcuuaucagacugau..	24	0	seq
.......uagcuuaucagacugGu..	3	1	seq
.......uagAuuaucagacugau..	1	1	seq
.......uagcuuaucagacugaug...	309	0	seq
.......uagcuuaucagacugaugu..	4619	0	seq
.......uagcuuaucagacugauguu...	8823	0	seq
.......uagcuuaucagacugauguug..	57380	0	seq
.......uagcuuaucagacugauguuga...	105327	0	seq
.......uagcuuaucagacugauguugac..	94505	0	seq
.......uagcuuaucagacugauguugacu...	20087	0	seq
.......uagcuuaucagacugauguugacug..	5	0	seq
.......uagcuuaucagacugauguugacugu.......................................	12	0	seq
.......uagcuuaucagacugauguugacuguu......................................	11	0	seq
........agcuuaucagacugaugu..	4	0	seq
........agcuuaucagacugauguu...	6	0	seq
........agcuuaucagacugauguug..	65	0	seq
........agcuuaucagacugauguuga...	88	0	seq
........agcuuaucagacugauguugac..	91	0	seq
........agcuuaucagacugauguugacu...	29	0	seq
.........gcuuaucagacugaugu..	1	0	seq
.........gcuuaucagacugauguug..	10	0	seq
.........gcuuaucagacugauguuga...	21	0	seq
.........gcuuaucagacugauguugac..	11	0	seq
.........gcuuaucagacugauguugacu...	5	0	seq
..........cuuaucagacugauguu...	1	0	seq
...........uuaucagacugauguug..	3	0	seq
...........uuaucagacugauguuga...	3	0	seq
...........uuaucagacugauguugac..	1	0	seq
...........uuaucagacugauguugaU..	1	1	seq

oar-miR-21

```
ugucggguagcuuaucagacugauguugacuguugaaucucauggcaacagcagucgaugggcugucugaca
............uaucagacugauguuga.........................................    2          0          seq
.............................................caacagcagucgaugggcug.......    2          0          seq
.............................................caacagcagucgaugggcugu......    9          0          seq
.............................................caacagcagucgaugggcuguc.....    7          0          seq
..............................................aacagcagucgaugggcugu......    1          0          seq
```
